# Neuro-Plastic Mechanisms of Pain and Addiction

**DOI:** 10.3390/ijms231810793

**Published:** 2022-09-16

**Authors:** Bae Hwan Lee, Hee Young Kim, Hee Kee Kim

**Affiliations:** 1 Department of Physiology, Yonsei University College of Medicine, Seoul 03722, Korea; 2 Brain Korea 21 PLUS Project for Medical Science, Yonsei University College of Medicine, Seoul 03722, Korea; 3 Department of Pain Medicine, The University of Texas MD Anderson Cancer Center, 1515 Holcombe Blvd., Houston, TX 77030, USA

Pain plays an important role in human survival. If acute pain progresses towards chronic pain, however, the pain often becomes unbearable and loses its survival value, as it begins to intefere with the patient’s life.

Over the last three decades, one of the most significant advancements in pain research has been the realization of neuro-plastic changes in the central nervous system (CNS) in chronic pain conditions [1]. In the spinal cord, central sensitization occurs when persistent nociceptive inputs enter the spinal cord. The sensitized dorsal horn neurons then show lowered stimulation thresholds and respond more vigorously to external stimuli [1,2]. This central sensitization is a form of plastic change and is believed to be the mechanism underlying chronic pain [3,4,5]. Although central sensitization has been most extensively studied in the spinal cord, similar plastic changes likely occur at multiple sites along the neuraxis from the periphery to the CNS [1]. In chronic pain induced by nerve injury, these changes occur in both sensory systems and reward systems, including the ventral tegmental area, nucleus accumbens (NAc), and forebrain areas [6,7]. The processes involved in the central sensitization are multiple and diverse. These processes include nociceptive neuronal hyper-excitability, which leads to hyperalgesia and allodynia.

In the CNS, changes in membrane receptors will lead to changes in drug sensitivity. In the cases of chronic pain, pain medications such as opioids have been most commonly prescribed and used. However, patients with repeated pain medications frequently experience drug dependence and addiction. Pain increases craving for opioids [8], and undertreated pain may lead to drug-seeking behaviors [9]. Detailed studies to elucidate the exact mechanisms involved in these processes are obviously required for addiction management and analgesic treatment scheme development regarding narcotics in chronic pain patients.

It has been suggested that pain and addiction may share common neurobiological mechanisms [10]. Thus, the purpose of this Special Issue was to unite experts from pain and addiction fields to investigate the neuro-plastic mechanisms of pain and addiction. Ten original articles were published in this Special Issue.

Neuropathic pain induced by injury or dysfunction of the peripheral nervous system (PNS), as well as CNS, can produce various sensory symptoms and cognitive disorders. Tyrtyshnaia et al. [11] found that nerve injury resulted in microglia activation in the contralateral hippocampus and synaptamide (N-docosahexaenoylethanolamine), an endogenous metabolite of docosahexaenoic acid, inhibited the activation of microglia and pain behaviors in mice. Synaptamide prevented memory impairment and hippocampal long-term potentiation (LTP) impairment [11]. These results suggest that synaptamide has a beneficial effect on synaptic plasticity and cognitive processes in the hippocampus of neuropathic mice. Similarly, trigeminal neuropathic pain leads to deficits in cognitive functions, such as decision-making in a gambling task of rats. A study by Murugappan et al. [12] revealed that blocking afferent signals with a tetrodotoxin (TTX) reduced mechanical allodynia and rescued decision-making deficits, but the TTX could not reduce the allodynia and improve decision-making deficits at a later phase, suggesting that early pain relief is more important for functional recovery from pain-related cognitive deficits. Using a CNS model of neuropathic pain, Lee et al. [13] observed that the level of superoxide marker dihydroethidium significantly increased in rats with spinal cord injuries compared to the sham rats. Furthermore, intrathecal administration of superoxide scavenger 4-hydroxy-2,2,6,6-tetramethylpiperidinyl-1-oxyl (TEMPOL) and non-specific reactive oxygen species (ROS) scavenger N-tert-butyl-α-phenylnitrone (PBN) decreased firing rates in the spinal cord injury (SCI) group. Intrathecal administration of pCaMKII inhibitor KN-93 or TEMPOL attenuated the mechanical sensitivity at the body trunk of the SCI group. These results indicate that the superoxide and pCaMKII pathways contribute to chronic neuropathic pain following SCI.

In relation to inflammatory pain, Kang et al. [14] reported that intrathecal treatment of capsazepine, a transient receptor potential vanilloid 1 (TRPV1) antagonist, significantly reduced mechanical allodynia and thermal hyperalgesia induced by carrageenan and phosphorylation of N-methyl-D-aspartate (NMDA) receptor subunit 2B (NR2B) in the spinal cord. The results suggest that inhibition of TRPV1 may attenuate inflammatory pain via NMDA receptors. According to Chun et al. [15], single or repeated treatments of BD1047—a prototypical sigma-1 receptor (Sig-1R) antagonist—not only reduced thermal and mechanical hyperalgesia, but also reduced CC-chemokine ligand 2 (CCL2) expression in dorsal rood ganglion (DRG) neurons and microglial activation in the spinal dorsal horn in the rat inflammatory pain models induced by an intraplanar injection of zymosan or an intra-articular injection of Complete Freund’s adjuvant (CFA). These results suggest that the analgesic effect of BD1047 in the inflammatory pain is mediated by the inhibition of CCL2 release in primary afferents and spinal microglia. They also indicate that sigma-1 receptor (Sig-1R) antagonist is a potential pharmacological agent in inflammatory pain. On the other hand, a study by Wang et al. [16] shows the effectiveness of a non-pharmacological intervention as a promising treatment strategy for alleviating pain. In order to explore the effects of low-intensity shock wave therapy (Li-ESWT) in an animal model of capsaicin-induced prostatitis, Wang et al. [16] conducted molecular and imaging studies. Intra-prostatic capsaicin injection increased cyclooxygenase-2 (COX-2), nerve growth factor (NGF), brain-derived neurotrophic factor (BDNF), tropomyosin receptor kinase A (Trk-A), and TRPV1 expressions in the DRG and the spinal dorsal horn, whose effects were significantly downregulated by Li-ESWT on the prostate. Following intra-prostatic injection of capsaicin, the activity of blood-oxygenation-level-dependent (BOLD) functional magnetic resonance imaging (fMRI) responses increased in brain regions related to pain, such as the caudate–putamen, periaqueductal gray, and thalamus, whose BOLD signals were reduced following Li-ESWT. These findings may suggest potential mechanisms of a non-pharmacological intervention Li-ESWT on modulation of prostatitis-induced pain.

Regarding addiction, several very interesting papers were published. In a trial to determine the role of the hypothalamus (LH) in mediating acupuncture effects on addiction, Ahn et al. [17] found that systemic injection of cocaine increased locomotor activity and reward-associated ultrasonic vocalizations, which were reduced by stimulation of needles inserted into the Shenmen point (HT7), but not the Zusanli (ST36) nor the Hegu (LI5). The effect of the acupuncture was diminished by chemical lesion of the LH. The results suggest that acupuncture stimulation at the HT7 acupoint recruits the LH to inhibit psychomotor responses following cocaine injection. According to Seo et al. [18], acupuncture stimulation at HT7 alleviated anxiety and ultrasonic vocalizations in rats which received repeated intraperitoneal administration of alcohol, increased the expressions of mature brain-derived neurotropic factor (mBDNF) and phosphorylated tropomyosin receptor kinase B (pTrkB) in the amygdala, and reduced corticotropin-releasing hormone (CRH) expression in the paraventricular nucleus. Using the intracranial self-stimulation (ICSS) paradigm in the medial forebrain bundle (MFB), Yoon et al. [19] found that acupuncture stimulation at HT7 produced a rightward shift of the frequency–rate curve and raised the ICSS thresholds. However, it did not affect the threshold-lowering effects of cocaine, indicating that acupuncture stimulation at HT7 effectively regulates the ICSS thresholds of the MFB in drug-free rats. These studies showed the beneficial effects of acupuncture as a non-pharmacological intervention and its underlying mechanisms in addiction management. Addiction is also closely associated with anxiety [20], and beta-adrenoceptors (β-ARs) in the medial prefrontal cortex (mPFC) have been implicated in the modulation of anxiety. Meanwhile, Lei et al. [21] observed that optogenetic stimulation of β2-ARs, a subtype of β-ARs, induced anxiety, reducing social interaction in mice. They also found that the knockdown of β2-ARs decreased anxious behavior and increased social interaction. These data suggest that β2-AR signaling in the mPFC plays a crucial role in anxiety, and inhibition of β2-AR signaling may be effective in treating anxiety.

The mechanisms of pain and addiction are highly complex. However, understanding the detailed neurobiological mechanisms underlying pain and addiction and their relationship could contribute to the development of efficacious, neural-mechanism-based strategies for the treatment of pain and addiction.

## Data Availability

Not applicable.
